# Strongly-motivated positive affects induce faster responses to local than global information of visual stimuli: an approach using large-size Navon letters

**DOI:** 10.1038/srep19136

**Published:** 2016-01-12

**Authors:** Yasuki Noguchi, Kouta Tomoike

**Affiliations:** 1 Department of Psychology, Graduate School of Humanities, Kobe University, Japan. 1-1 Rokkodai-cho, Nada, Kobe, 657-8501, Japan

## Abstract

Recent studies argue that strongly-motivated positive emotions (e.g. desire) narrow a scope of attention. This argument is mainly based on an observation that, while humans normally respond faster to global than local information of a visual stimulus (global advantage), positive affects eliminated the global advantage by selectively speeding responses to local (but not global) information. In other words, narrowing of attentional scope was indirectly evidenced by the elimination of global advantage (the same speed of processing between global and local information). No study has directly shown that strongly-motivated positive affects induce faster responses to local than global information while excluding a bias for global information (global advantage) in a baseline (emotionally-neutral) condition. In the present study, we addressed this issue by eliminating the global advantage in a baseline (neutral) state. Induction of positive affects under this state resulted in faster responses to local than global information. Our results provided direct evidence that positive affects in high motivational intensity narrow a scope of attention.

Much research has investigated a relationship between emotion and attention. One of hot topics in these days is how one’s affective states influence his/her scope of attention (narrowing or broadening). Earlier studies have examined this issue by focusing on a valence (positive-negative dimension) of emotion. Those studies indicated that positive affects (e.g. happiness) broadened one’s attentional scope whereas negative affects (e.g. sadness) narrowed it^1^,^2^,^3^,^4^,^5^. On the other hand, recent studies focused on another dimension of emotion: intensity. Especially, they focused on a motivational intensity of emotion, an urge to move toward (or move away from) a stimulus. It was argued that one’s attentional scope was not modulated by valence (positive or negative) but by the motivational intensity (high or low) of emotion. Affective states in high motivational intensity (e.g. desire, enthusiasm, and disgust) cause narrowing of attentional scope, while affective states in low motivational intensity (e.g. sadness and satisfaction) cause broadening^6^.

An effective approach discriminating those two theories (valence vs. motivational intensity) is to test a situation where the two theories predict different consequences. For example, what happens to one’s scope of attention (narrowing or broadening) when positive affects in high motivational intensity is induced? We will focus on this case in the present study. If attentional scope is modulated by emotional valence, the positive affects would broaden attentional scope of subjects (as predicted by the earlier studies). If intensity is critical, however, the high-intensity emotions would narrow attentional scope even though they are positive in valence. A representative study investigating this point was performed by Gable and Harmon-Jones^7^. They presented subjects with two visual stimuli in each trial: prime and target. The prime was a picture of objects to manipulate affective states of subjects. In an experimental condition, pictures of desserts were shown to evoke positive affects in high-motivational intensity, while pictures of rocks (emotionally-neutral stimuli) were used in a control condition. After viewing the prime for 6 s, subjects were presented with another stimulus (target) on which they performed the Navon letter task^8^. The stimulus in this task (Navon letter) was a large letter composed of smaller letters (see Fig. 1A for examples). Subjects were asked to identify a target letter (T or H) embedded in either global or local dimension of the Navon letter. A point was that they needed to zoom in their attentional scope to identify the small (local) letters but had to zoom out to identify the large (global) letter. Faster responses to large target letters (T or H) thus indicated a global (broad) focus of attention, while faster responses to small targets indicated a local (narrowed) focus. In the neutral-prime condition, researchers observed faster responses to global than local targets. This precedence of global information, called “global advantage”, is a typical phenomenon seen in the Navon letter task^9^,^10^,^11^,^12^,^13^,^14^,^15^,^16^,^17^. Viewing the dessert primes, however, shortened reaction times to local targets, while no significant change in reaction times were observed for global targets. Consequently, the reaction times to local and global targets were comparable in the dessert-prime condition (a diminishment of global advantage, see Fig. 1B). Based on those results, Gable and Harmon-Jones (2008) concluded that positive affects in high motivational intensity narrowed attentional scope, providing evidence that attention is modulated by intensity, not valence, of emotions.

Although this study was well-designed, a potential problem would be a global advantage in the baseline (neutral-prime) condition. Longer reaction times to local than global targets in the baseline condition suggest that task difficulty was not balanced between local- and global-target trials. The selective shortening of reaction times to local targets (by dessert primes) might result from this difference in task difficulty (e.g. ceiling effect of reaction times in global-target trials). It is therefore preferable that the reaction times to local and global targets are balanced in the neutral-prime condition. Another problem in Gable and Harmon-Jones (2008) would be a diminishment of global advantage in dessert-prime condition. Although this diminishment might reflect a selective shortening of reaction times to the local target, another interpretation is that the positive affects eliminated perceptual (or attentional) bias for the global information in the baseline condition. In this latter interpretation, an effect of dessert primes was not narrowing of attentional focus but an equal assignment of attentional resources into local and global letters. To demonstrate a selective facilitation of the processing of local but not global information, it is necessary to show directly that positive affects enabled faster responses to local than global information of Navon letters (local advantage) while excluding global advantage in a baseline condition.

Two potential problems indicated above highlight an importance of balanced difficulty of global- and local-target trials in a baseline condition. Indeed, Nittono *et al.* (2012) conducted a visual search task to which this problem of balanced difficulty was not applicable. In this task, subjects were presented with a matrix (4 rows × 10 columns) of 40 digits (0–9). They were asked to search the matrix for a target digit (e.g. 3) and to write down the number of counts on a separate sheet. The authors found that viewing images of pleasant food induced no change in task performances despite a requirement of narrowed attentional focus in this task^18^. Their results thus might suggest a possibility that the shortened reaction times to local targets in Gable and Harmon-Jones (2008) did *not* reflect narrowing of attentional scope by dessert primes.

In the present study, we addressed this issue by eliminating global advantage of the Navon letter task in a baseline (neutral-prime) condition. As shown in Fig. 1C, the Navon letters in our main condition (large-letter condition) were larger in size and composed of smaller number of local letters than previous studies. Furthermore, those local letters were variable in their sizes and spaced irregularly. Those manipulations hamper perceptual grouping of local letters and the processing of global information of the Navon letters^19^, which would eliminate global advantage in a neutral-prime condition. If the highly-motivated positive affects truly narrow a scope of attention, this should be observed in the present study as faster reaction times to local than global targets (local advantage) in a dessert-prime condition, rather than a diminishment of global advantage in previous studies.

## Results

**Figure 1:**
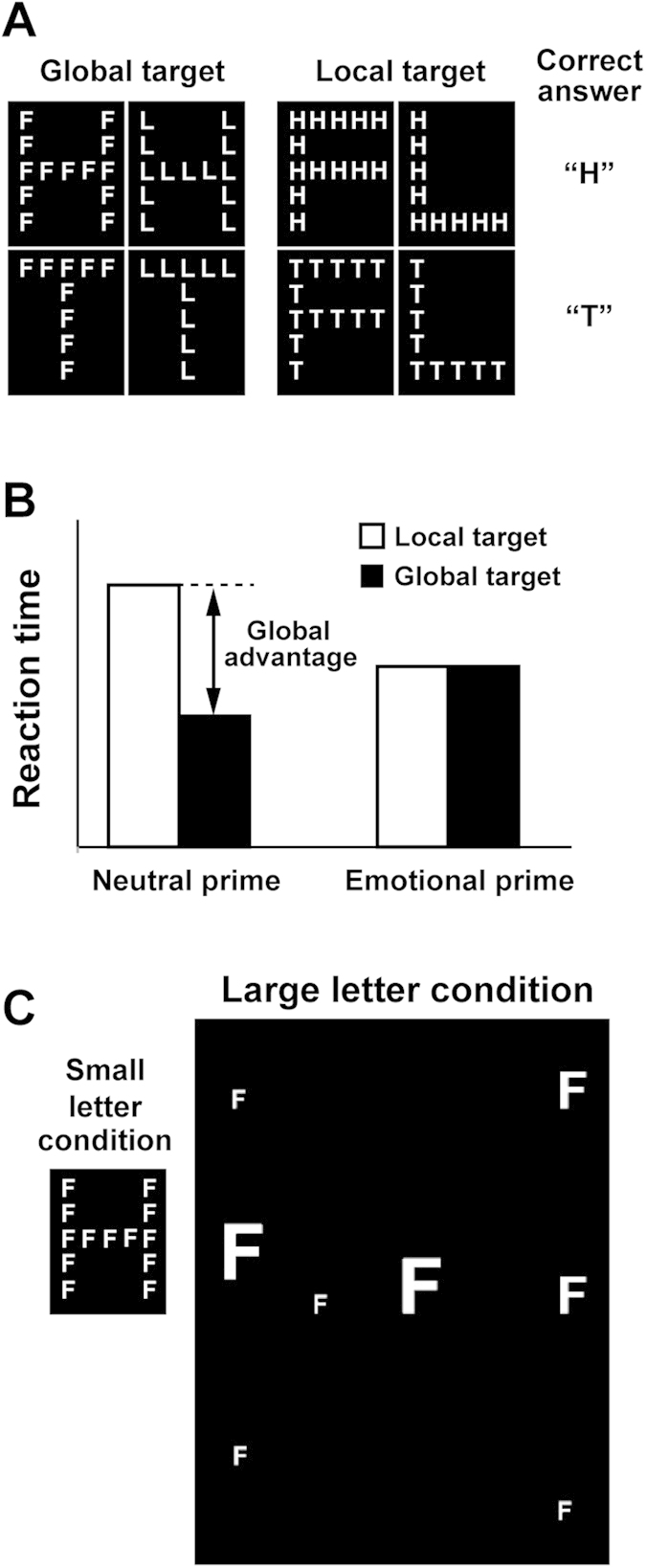
Stimuli and task. (**A**) Eight types of Navon letters used in the present study. Subjects identified T or H embedded either in global or local level of a Navon letter. They pressed one button to a large (global) T or small (local) Ts and another button to a large H or small Hs as quickly as possible. (**B**) Global advantage in reaction times. It is known that humans normally respond faster to global than local information of visual stimulus (global advantage). Previous studies showed narrowing of attentional scope by emotional changes through a diminishment of global advantage; while observers responded faster to global targets of Navon letters in an emotionally-neutral (neutral-prime) condition, an induction of strong affects by emotional primes selectively shortened the reaction times to local targets, resulting in a diminishment of the global advantage. (**C**) Navon letters in small-letter and large-letter conditions in the present study. After viewing a prime (either neutral or emotional prime, see Methods for details) for 6 s, subjects discriminated whether a Navon letter (either in small or large size, randomly intermixed) had T(s) or H(s). A Navon letter in the large-letter condition was designed to eliminate the global advantage in neutral-prime condition, because of its difficulty in perceiving a global shape.

In every trial, participants viewed two stimuli (prime and target) sequentially presented at the center of a screen. Primes for a control condition (neutral primes) were pictures taken from the International Affective Picture System (IAPS)^20^, while primes in an experimental condition were images of desserts. After viewing a prime for 6 s, subjects performed the Navon letter task (T or H) on a target (Fig. 1A). A magnitude of the global advantage was experimentally manipulated between small-letter and large-letter trials^19^. In the small-letter trials, a size of a global letter was 2.375 × 3.125 deg, and each stroke of a global letter was made up of five local letters closely and equally spaced (Fig. 1C, left). In contrast, a global letter in the large-letter trials was much larger (9.5 × 12.5 deg) consisting of a smaller number of local letters (in variable sizes) irregularly spaced (Fig. 1C, right). Crossing two types of prime images (neutral/dessert) with sizes of global letter (small/large) and dimensions of target (global/local) produced 8 conditions. Subjects performed 9 sessions of 32 trials in which trials of those 8 conditions were randomly intermixed. After completing all sessions, they also performed an emotional rating of all prime images used in the experiment. For each of 288 primes (144 neutral and 144 dessert), they rated its emotional valence (negative-positive) and arousal (relaxed-excited) using a 5-point scale.

### Emotional rating for primes

**Figure 2:**
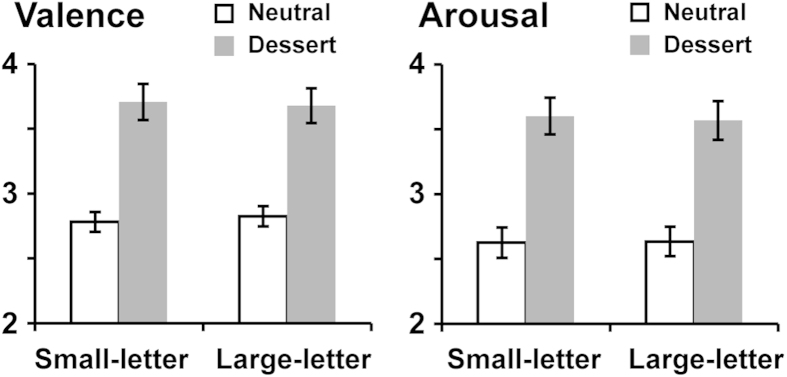
Scores of an emotional rating. After completing all sessions (288 trials) of the Navon-letter task, subjects rated emotional valence (1: negative-5: positive) and arousal (1: relaxed-5: excited) for the 288 primes. Scores for dessert (emotional) primes were higher than those for neutral primes both in small- and large-letter conditions, indicating that positive affects in high motivational intensity was successfully induced by dessert primes. From this and subsequent figures, errors bars denote SE across subjects.

Figure 2 shows results of an emotional rating for neural and dessert primes. In the valence rating (1: negative-5: positive, left panel of Fig. 2), scores for dessert primes were higher than those for neutral primes both in small-letter and large-letter trials. A two-way repeated-measures ANOVA of prime (neutral/dessert) and target size (small/large) indicated a significant main effect of prime (dessert > neutral, *F*(1, 17) = 29.4, *p* < 0.001, partial *η*^2^ = 0.63) as well as an interaction (*F*(1, 17) = 4.82, *p* = 0.042, *η*^2^ = 0.22). No main effect of letter size was observed (*F*(1, 17) = 0.15, *p* = 0.70, *η*^2^ = 0.01). The significant interaction mainly resulted from lower valence scores for neutral primes in the small-letter (2.78 ± 0.08, mean ± SE) than large-letter (2.82 ± 0.08) conditions. Since no significant difference was observed in a direct comparison between them (*t*(17) = 1.96, *p* = 0.07), we presume that valence of neutral primes was overall balanced between the small- and large-letter conditions.

Scores of the arousal rating (1: relaxed-5: excited) showed similar results (right panel of Fig. 2). A two-way repeated-measures ANOVA yielded a significant main effect of prime (dessert > neutral, *F*(1, 17) = 19.1, *p* < 0.001, *η*^2^ = 0.53), but not for a main effect of letter size (*F*(1, 17) = 0.31, *p* = 0.59, *η*^2^ = 0.02) or interaction (*F*(1, 17) = 1.34, *p* = 0.26, *η*^2^ = 0.07). Those results of emotional rating overall suggested that, although we used different sets of prime images between small-letter and large-letter trials, dessert primes evoked highly-motivated positive affects compared to neutral primes in both types of trials.

### Reaction times for targets

**Figure 3:**
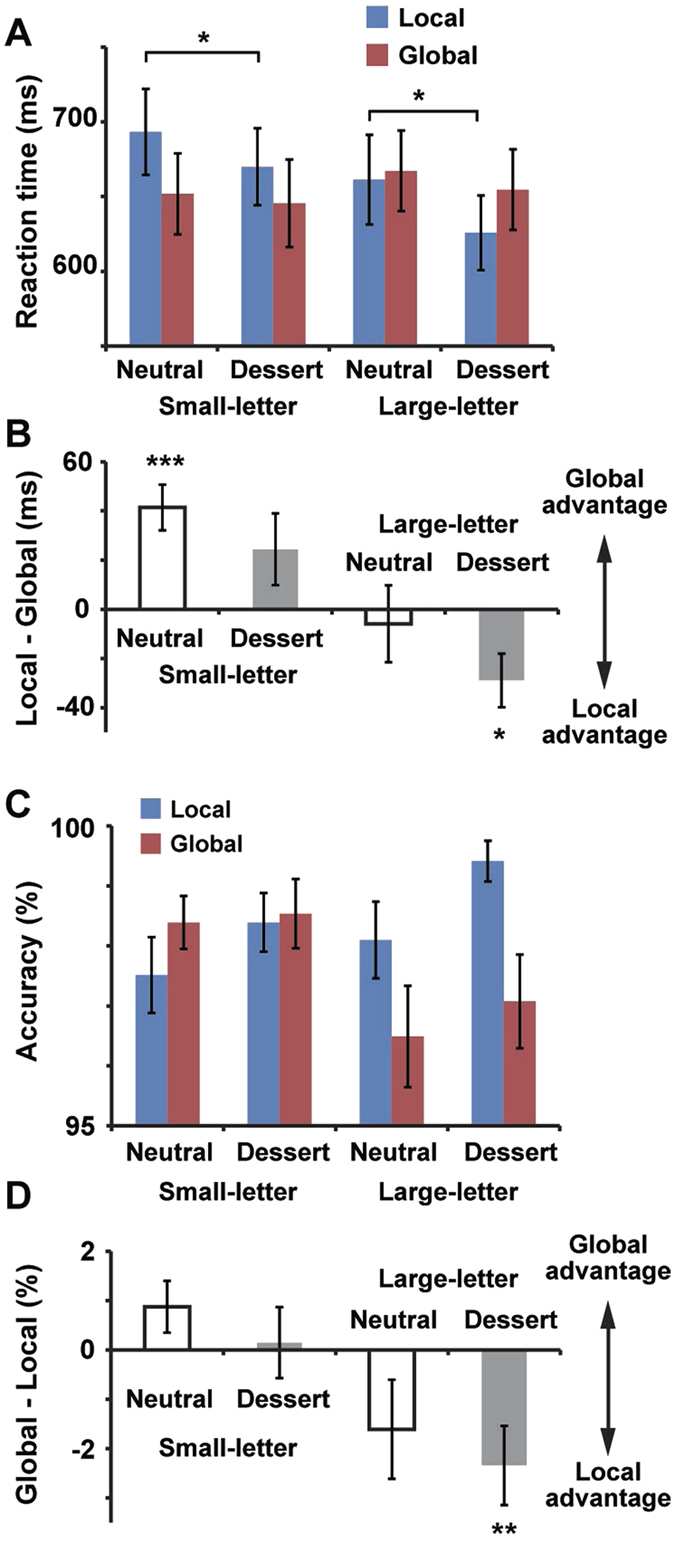
Reaction times and accuracy in the Navon letter task. (**A**) Reaction times in all 8 conditions. (**B**) Differential reaction times (local target minus global target) for each of four (neutral/dessert × small-letter/large-letter) conditions. In small-letter condition, subjects responded faster to global than local targets (global advantage) in neutral-prime trials, although the global advantage was diminished in dessert-prime condition. On the other hand, reaction times to large letters were comparable between global- and local-targets trials in neutral-prime condition. This elimination of the global advantage in a control condition produced a local advantage (faster responses to local than global target) in dessert-prime condition. (**C**) Task accuracy in 8 conditions. (**D**) Differential accuracy between global- and local-target trials. Consistent with the reaction times (**B**), local advantage was observed when dessert primes were followed by large-letter targets. *p < 0.05, **p < 0.01, ***p < 0.001.

We then analyzed reaction times of the Navon letter task (mean ± SE across 19 subjects, Fig. 3A,B). To inhibit an effect of outliers, we computed median reaction times (not mean reaction times) for each condition of each subject. A three-way repeated-measures ANOVA of prime (neutral/dessert), letter size (small/large) and target (global/local) indicated a significant main effect of prime (neutral > dessert, *F*(1, 18) = 19.9, *p* < 0.001, *η*^2^ = 0.53) as well as an interaction of letter size and target (*F*(1, 18) = 18.0, *p* < 0.001, *η*^2^ = 0.50). No other main effect or interaction was obtained (*F* < 4.06, *p* > 0.05 for all). Since we observed the main effect of prime, reaction times in neutral- and dessert-prime trials were separately analyzed using two-way repeated-measures ANOVAs of letter size (small/large) and target (global/local). In neutral-prime trials, the ANOVA showed non-significant main effect of letter size (*F*(1, 18) = 0.97, *p* = 0.34, *η*^2^ = 0.05) and target (*F*(1, 18) = 3.37, *p* = 0.08, *η*^2^ = 0.16) but a significant interaction between those two factors (*F*(1, 18) = 7.84, *p* = 0.012, *η*^2^ = 0.30). Post-hoc comparisons indicated a significant (*p* = 0.0003) difference between global- and local-targets trials only in small-letter condition. The size × target interaction above therefore shows that the global advantage in small-letter condition was successfully eliminated in large-letter condition. We then analyzed reaction times in dessert-prime trials. The two-way ANOVA revealed a significant main effect of letter size (*F*(1, 18) = 5.40, *p* = 0.032, *η*^2^ = 0.23) and size × target interaction (*F*(1, 18) = 7.42, *p* = 0.014, *η*^2^ = 0.29) with a non-significant main effect of target (*F*(1, 18) = 0.07, *p* = 0.79, *η*^2^ = 0.004). Post hoc-tests indicated a significant difference between global- and local-target trials (global > local, *p* = 0.017) only in large-letter condition. Those data thus showed that subjects responded faster to the local than global targets (local advantage) in dessert-prime trials of large-letter condition.

Figure 3A shows a bar graph of reaction times in all eight conditions that directly compared neutral- and dessert-prime trials. In small-letter condition (left half of Fig. 3A), dessert primes significantly shortened the reaction times to local targets (blue bars, *t*(18) = 2.25, *p* = 0.037, *d* = 0.20) whereas no shortening was observed for global targets (red bars). This diminishment of global advantage by the dessert primes was identical to a previous study^21^. In large-letter condition (right half of Fig. 3A), the global advantage in neural-prime trials was eliminated because of difficulties in the processing of global letter information. This lack of global advantage in a baseline (neural-prime) condition enabled a fair comparison between global- and local-target trials, resulting in the local advantage in dessert-prime conditions (see also Fig. 3B in which differential reaction times, local – global, were displayed). Our data of reaction times therefore indicated that dessert primes caused narrowing of attentional focus regardless of whether the global advantage was present or not in a control condition.

### Task accuracy

Figure 3C shows accuracy of the Navon letter task in the eight conditions. A three-way repeated-measures ANOVA of prime (neutral/dessert), letter size (small/large) and target (global/local) yielded a significant main effect of target (local > global, *F*(1, 18) = 4.68, *p* = 0.044, *η*^2^ = 0.206) as well as an interaction of letter size and target (*F*(1, 18) = 12.9, *p* = 0.002, *η*^2^ = 0.417). No other main effect or interaction was obtained (*F* < 4.26, *p* > 0.05 for all). Those results indicate that a use of large Navon letters prevented the processing of global information but facilitated the processing of local information of stimuli, which was consistent with the reaction times (the lack of global advantage in the large-letter condition, Fig. 3A,B). As shown in differential (global – local) accuracy for each of four (neutral/dessert × small-letter/large-letter) conditions (Fig. 3D), this local advantage was especially strong and reached significance when dessert primes were followed by large-letter targets (*t*(18) = 2.92, *p* = 0.009, *d* = 0.89, one-group *t*-test). Those results in task accuracy were overall consistent with the reaction times (Fig 3B), showing that main results in reaction times (an emergence of local advantage by the dessert primes) did not reflect a speed-accuracy trade-off.

## Discussion

In the present study, we investigated how strongly-motivated positive affects influenced a scope of attention. A use of large Navon letters (large-letter condition) eliminated a perceptual bias favoring the processing of global information in control condition, which enabled a fair comparison of reaction times between global- and local-target trials. Results revealed faster responses to local than global targets in dessert-prime trials, providing direct evidence that positive affects in high motivational intensity speeded recognition of local letters by narrowing a scope of attention.

The Navon letter task in the present study is a commonly-used measure of local/global processing because it has some advantages over other tasks. First, the Navon letter task can directly gauge an effect of emotional changes on recognition (perception) of stimuli. For example, a previous study^22^ measured attentional scope by assessing recognition memory of subjects for neutral words presented at the center or periphery of a monitor. Performances for centrally-presented words were better after pre-goal (high-intensity) positive affect cues than after post-goal (low-intensity) positive affect cues. Although those results in the memory task indicated that highly-motivated positive affects narrowed attentional focus, it remained unclear whether the positive affects facilitated perception or memory for the centrally-presented word. This problem is not applicable to the Navon letter task, because no memorization of target stimuli is required. Second, a task rule is clear and participants seek for a sole answer (H or T) in ever trial. This can eliminate an ambiguity in other tasks (e.g. a matching or similarity-judgment task^23^) where no decisive answer is present. Finally, the Navon letter task assesses a scope of attention based on two different measures: reaction time and task accuracy. Converging results of reaction times (Fig. 3A,B) and accuracy (Fig. 3C,D) therefore provide strong evidence that highly-motivated positive affects enabled both faster and more accurate responses to local than global information of a visual stimulus.

One limitation of the present study was that we tested only a situation when primes induced highly-motivated positive affects. No comparison between strong positive vs. weak positive affects was performed. If attentional scope was modulated by the motivational intensity, results in previous studies^22^,^24^ predict that an induction of weak (rather than strong) positive emotions broadens scope of attention. This would selectively decrease the reaction times to global targets, producing the global advantage in an experimental (affective prime) condition even when the large Navon letters are used. Another interesting approach is to test an effect of negative (not positive) emotions on attention. Using the Navons letter task, a previous study showed that negative affects in low motivational intensity (e.g. sadness) induced broadening of attentional scope while those in high motivational intensity (e.g. disgust) caused narrowing^21^. Those findings would be further strengthened if the global advantage in their neutral-prime conditions is successfully removed by large Navon letters used in the present study.

## Methods

### Subjects

Twenty healthy subjects (9 females, mean and SD of ages: 20.9 ± 1.3) participated in the present study. All participants had normal or corrected-to-normal vision. Data of one subject were excluded from analyses because he showed task accuracy lower than a 2SD range of the 20 participants (93.1–100%). Informed consent was received from each subject after the nature of the study had been explained. All experiments were carried out in accordance with guidelines and regulations approved by the ethics committee of Kobe University, Japan.

### Procedures

All visual stimuli were generated using the MATLAB Psychophysics Toolbox^25^,^26^ and presented on a CRT monitor (resolution: 1024 × 768 pixels, refresh rate: 60 Hz). In every trial, participants viewed two stimuli sequentially presented at the center of a screen: prime and target. The prime was a picture of objects or a scene that was meant to manipulate affective states of subjects. Primes for a control condition (neutral primes) were taken from the International Affective Picture System (IAPS)^20^ and consisted of commodities (e.g. chair), geometric shapes, and ordinary scenes (e.g. street), etc. Scores of valence and arousal ratings^20^ averaged across all neutral primes were 5.2 (valence, 1: negative – 9: positive) and 3.3 (arousal, 1: relaxed – 9: excited), indicating that those primes were emotionally neutral and non-stimulating. On the other hand, we presented images of desserts as primes in an experimental condition to evoke approach-motivated positive affects of observers^7^. Those images were collected from the Internet and cropped into the same size as the neutral primes (visual angle: 16 × 12 deg). To eliminate a possibility that non-emotional aspects (e.g. low-level visual features) of the primes influenced affective states of subjects, we controlled luminance and root-mean-square (RMS) contrast of the primes between the neutral and dessert conditions. Statistical comparisons indicated non-significant difference in the luminance (*t*(286) = 0.05, *p* = 0.96, Cohen’s *d* = 0.006) and RMS contrast (*t*(286) = 1.26, *p* = 0.21, *d* = 0.15) between the neutral and dessert primes.

After viewing a prime for 6 s, subjects performed a perceptual judgment on a target (Navon letter). As shown in Fig. 1A, we presented eight types of target stimuli randomly intermixed across trials. Subjects were asked to indicate whether a target contained a letter T or H either in a global or local level. Global targets were those in which a large T or H was made of smaller Ls or Fs, and local targets were those in which a large L or F consisted of smaller Ts or Hs. Subjects pressed one button as quickly as possible when they found T (a large T or small Ts) and pressed another when they found H (a large H or small Hs). Faster responses to large than small targets indicated a global focus of attention, whereas faster responses to small targets indicated a local focus^7^. As described in Introduction, a magnitude of the global advantage was experimentally manipulated between small-letter and large-letter trials^19^. In the small-letter trials, a size of a global letter was 2.375 × 3.125 deg, and each stroke of a global letter was made up of five local letters closely and equally spaced (Fig. 1C, left). In contrast, a global letter in the large-letter trials was much larger (9.5 × 12.5 deg) consisting of a smaller number of local letters (in variable sizes) irregularly spaced (Fig. 1C, right). While the global H, T, F, and L in the small-letter condition were made up of 13, 9, 13, and 9 local letters, respectively, they consisted of 8 (H), 6 (T), 8 (F), and 6 (L) local letters in the large-letter condition. Stimulus parameters in the large letter condition (sizes of global and local letters and numbers of local letters) were determined based on results in our pilot study using different group of subjects. We expected faster responses to global than local targets in the small-letter trials, while no global advantage would be observed in the large-letter trials because of difficulties in the processing of global information.

Each trial started with a fixation screen of 0.5 s, followed by a prime (neutral or dessert, 6 sec). After another fixation of 0.5 s, a Navon letter (small or large) appeared until subject pressed any key. Crossing two types of prime images (neutral/dessert) with sizes of global letter (small/large) and dimensions of target (global/local) produced 8 conditions. Subjects performed 9 sessions of 32 trials in which trials of those 8 conditions were randomly intermixed. After completing all sessions, they also performed an emotional rating of all prime images used in the experiment. For each of 288 primes (144 neutral and 144 dessert), they rated its emotional valence (negative-positive) and arousal (relaxed-excited) using a 5-point scale. A presentation order of those primes was identical to that in the main sessions with the Navon letter task.

## Additional Information

**How to cite this article**: Noguchi, Y. and Tomoike, K. Strongly-motivated positive affects induce faster responses to local than global information of visual stimuli: an approach using large-size Navon letters. *Sci. Rep.* **6**, 19136; doi: 10.1038/srep19136 (2016).
